# Glyoxalase I Assay as a Possible Tool for Evaluation of Biological Activity of Antioxidant-Rich Plant Extracts

**DOI:** 10.3390/plants12051150

**Published:** 2023-03-03

**Authors:** Maura Nicoletta Laus, Federica Blando, Mario Soccio

**Affiliations:** 1 Department of Agriculture, Food, Natural Resources and Engineering (DAFNE), University of Foggia, Via Napoli, 25, 71122 Foggia, Italy; 2 Institute of Sciences of Food Production, CNR, Via Prov.le Lecce-Monteroni, 73100 Lecce, Italy

**Keywords:** antioxidant capacity, black carrot, durum wheat grain, glyoxalase I, Polignano carrot, ‘Sun Black’ tomato

## Abstract

The health-promoting properties of natural plant bioactive compounds are mainly attributable to their ability to counteract oxidative stress. This is considered a major causative factor in aging and aging-related human diseases, in which a causal role is also ascribed to dicarbonyl stress. This is due to accumulation of methylglyoxal (MG) and other reactive dicarbonyl species, leading to macromolecule glycation and cell/tissue dysfunction. The glyoxalase (GLYI) enzyme, catalyzing the rate-limiting step of the GSH-dependent MG detoxification pathway, plays a key role in cell defense against dicarbonyl stress. Therefore, the study of GLYI regulation is of relevant interest. In particular, GLYI inducers are important for pharmacological interventions to sustain healthy aging and to improve dicarbonyl-related diseases; GLYI inhibitors, allowing increased MG levels to act as proapoptotic agents in tumor cells, are of special interest in cancer treatment. In this study, we performed a new in vitro exploration of biological activity of plant bioactive compounds by associating the measurement of their antioxidant capacity (AC) with the evaluation of their potential impact on dicarbonyl stress measured as capability to modulate GLYI activity. AC was evaluated using TEAC, ORAC, and LOX-FL methods. The GLYI assay was performed using a human recombinant isoform, in comparison with the recently characterized GLYI activity of durum wheat mitochondria. Different plant extracts were tested, obtained from plant sources with very high phytochemical content (‘Sun Black’ and wildtype tomatoes, black and ‘Polignano’ carrots, and durum wheat grain). Results showed high antioxidant properties of the tested extracts, associated with different modes (no effect, activation, and inhibition) and effectiveness in modulating both GLYI activity sources. Overall, results indicate the GLYI assay as an advisable and promising tool for researching plant foods as a source of natural antioxidant compounds acting as GLYI enzymatic regulators to be used for dietary management associated the treatment of oxidative/dicarbonyl-promoted diseases.

## 1. Introduction

Plants synthesize and accumulate several chemically and structurally different secondary metabolites, known as phytochemicals. Some of these compounds occur constitutively, while others are generated in response to both abiotic and biotic stresses [1]. Almost all phytochemicals have excellent antioxidant potential, showing the capability to counteract reactive oxygen species (ROS), via both direct and indirect antioxidant mechanisms [2]. During the last few decades, the antioxidant potential of plant bioactive compounds has received a great deal of attention within biological and medical fields. In fact, oxidative stress, due to an increase in ROS steady-state levels resulting in damage to cell macromolecules, has been identified as a major causative factor in aging processes and in the pathogenesis of several aging-related human diseases [1,2,3]. In addition, it is widely accepted that supplementation with exogenous antioxidants or boosting of endogenous antioxidant defenses of the body can exert a beneficial role in extending and/or improving human health by counteracting the undesirable effects of oxidative stress [1].

It should be outlined that increased oxidative stress in aging and disease may boost another type of metabolic stress, known as dicarbonyl stress [4]. In addition, according to a vicious cycle, oxidative stress can result from dicarbonyl stress [5]. This stress is due to an abnormal accumulation of reactive dicarbonyl species (RCS) as a consequence of their increased production and/or decreased detoxification. RCS are constantly and unavoidably generated as byproducts of carbohydrate, amino-acid, and fatty-acid metabolisms [6,7]. In particular, the α-keto-aldehyde methylglyoxal (MG), representing the most common and physiologically relevant RCS, is mainly generated by the glycolytic and pentose phosphate pathways, as well as by the Calvin–Benson cycle in plant cells [6]. Due to its highly reactive nature, similarly to ROS, MG mainly targets protein residues, nucleic acids, and phospholipids leading to their inactivation and increased modification by the formation of advanced glycation end-products (AGEs) [8]. Increasing studies are corroborating the causal relationship between the accumulation of MG-derived AGEs in cell/tissue dysfunction, unveiling a causal role of dicarbonyl stress in determining healthy or unhealthy aging, as well as aging-related and oxidative-based diseases [4,6,7]. Dicarbonyl stress may be a mediator of obesity, insulin resistance, and diabetic microvascular complications, as well as cardiovascular disease, renal failure, and neurodegenerative disorders [7]. Moreover, dicarbonyls and AGEs may play a role in cancer onset and progression, since they evoke tumor-promoting inflammation and enhance proliferation, invasion, metastasis, angiogenesis, and apoptosis evasion of cancer cells [9].

The MG-induced and aging-related decline in the functional properties of cells, tissues, and whole organs starts from the perturbation of some crucial biochemical cellular processes, including mitochondrial function and detoxifying systems [4,10]. Mitochondrial proteins are a major target of dicarbonyl glycation [4], leading to an impaired functioning of mitochondrial respiratory chain. This is also associated with an increased ROS production, as well as a deactivation of antioxidant enzymes, thus resulting in an enhanced oxidative stress at mitochondrial level [4].

Under normal physiological conditions, steady-state concentrations of MG and other RCS are maintained at very low tolerable levels mainly thanks to the glyoxalase enzymatic network. This comprises two enzymes, glyoxalase I (EC 4.4.1.5, GLYI), and glyoxalase II (EC 3.1.2.6, GLYII), which catalyze the conversion of MG to the non-toxic D-lactate via the intermediate S-D-lactoylglutathione (S-LG), employing reduced glutathione (GSH) as a catalyst. In particular, GLYI catalyzes the isomerization to S-LG of hemithioacetal (HA), formed spontaneously from MG in the presence of GSH; then, S-LG is hydrolyzed by GLYII to D-lactate, regenerating GSH consumed in the GLYI-catalyzed step [7]. Under normal physiological conditions, the glyoxalase pathway metabolizes more than 99% of MG, thus representing the major route for MG detoxification and the primary defense against dicarbonyl stress-driven cell/tissue damage. The rate-limiting step for MG detoxification by the glyoxalase pathway is represented by the GLYI reaction; hence, the study of regulatory mechanisms of this enzyme is of very relevant interest. In this regard, over the past several decades, mammalian GLYI has increasingly been the subject of clinical research for its huge therapeutic potential [11]. In particular, the discovery and development of small-molecule regulators able to increase GLYI gene expression/activity have received growing interest for new pharmacological interventions aimed at maintaining healthy aging, as well as at improving the dicarbonyl-related diseases and complications [7,12,13]. On the other hand, it is known that increased expression/activity of GLYI can support the viability of tumor cells with high glucose uptake and glycolytic rates, by ensuring a high MG detoxification rate and preventing MG-induced apoptosis [12]. Therefore, the study of GLYI inhibition has also increasingly received special attention for anticancer drug development [12].

In light of the above explained close link between oxidative and dicarbonyl/glycation stress, antioxidant compounds appear to be promising agents for the prevention of AGE formation. Therefore, the study of the potential antiglycative activities of naturally occurring bioactive compounds with antioxidant properties is of particular interest [5]. In this study, we propose a new strategy for the in vitro exploration of biological activity of plant foods. It associates in vitro assessments of antioxidant capacity (AC) of plant extracts with the evaluation of their potential effect on dicarbonyl/glycation stress measured in terms of capability to affect RCS scavenging by modulating in vitro GLYI enzymatic activity.

We chose a commercially available and highly purified human recombinant isoform (h-GLYI) as a system for studying modulation of GLYI activity by phytochemicals. Since mitochondria are a major target of deleterious MG-induced dicarbonyl glycation, parallel assessment of the phytochemical effect on the activity of the highly purified mitochondrial fraction obtained from durum wheat (DWM) was also carried out. In fact, in these mitochondria, for the first time in a eukaryotic organism, a high GLYI activity was recently demonstrated and characterized (DWM-GLYI), being inhibited by curcumin and quercetin [14].

To investigate the effect of different phytochemicals on GLYI enzymatic activity, for the first time in this study, complex mixtures of natural bioactive compounds with antioxidant properties were evaluated after being extracted from different plant foods, including cereal grains (durum wheat whole meal) and fresh vegetables (tomato and carrot). In particular, vegetables very rich in anthocyanins were chosen, including the intensely skin-purple ‘Sun Black’ tomato genotype (compared to the corresponding wildtype tomato), as well as the black carrot and the purple Polignano carrot landrace of Apulia region. ‘Sun Black’ tomato is a recently bred tomato biosynthesizing anthocyanins in the peel, leading to an unusual purple skin color [15]. Unlike commercial carrots, black and Polignano carrots contain anthocyanins in addition to carotenoids. Black carrot has been bred to be uniform in terms of anthocyanin content of the taproot; instead, Polignano carrot is a landrace with various phenotypes, more or less rich in anthocyanins [16].

## 2. Results

### 2.1. Antioxidant Capacity Evaluation of Plant Food Extracts

In order to ascertain the antioxidant properties of the plant extracts under study, in vitro AC was investigated using the lipoxygenase-fluorescein (LOX-FL) method, based on secondary reactions between the soybean LOX-1 isoenzyme and fluorescein (FL) and able to provide a simultaneous estimation of different antioxidant mechanisms [17,18]. For comparison, parallel AC assessment was carried out with the well-established oxygen radical absorbance capacity (ORAC) [19] and Trolox equivalent antioxidant capacity (TEAC) [20] assays, measuring mainly the scavenging capacity against peroxyl and ABTS^●+^ radicals according to hydrogen atom (HAT) and single electron (SET) transfer mechanisms, respectively.

For AC evaluation, different extraction procedures were applied to the tested plant foods aimed at obtaining extracts enriched in different categories of antioxidant molecules (see Section 4). In particular, the non-solvent-extractable phenolic acid component bound to insoluble cell-wall polymers was obtained from durum wheat whole grain via alkaline hydrolysis of whole flour [21]. The polyphenolic component was extracted from the purple-colored anthocyanin-rich ‘Sun Black’ tomato (and the wildtype for comparison), as well as from black and Polignano carrots, using aqueous acidified methanol/ethanol able to stabilize anthocyanins on their flavylium forms [15]. Moreover, lipophilic antioxidant compounds were extracted from both wildtype tomato and durum wheat grain. In our previous studies, the antioxidant content of these extracts was analyzed in detail [15,16,21]. In particular, the most abundant phenolic compound in the insoluble-bound phenolic extract from durum wheat grain was ferulic acid (102–185 μmol/100 g dry weight, d.w.) [21]. The lipophilic extract from durum wheat showed a high carotenoid content, with lutein as the most abundant one (250–294 μg/100 g d.w.), as well as a very high tocol content, with β-tocotrienol as the most abundant one (3.55–3.68 mg/100 g d.w.) [21]. Phenolic extracts from ‘Sun Black’ tomato have already been characterized by a high total anthocyanin content in the peel, giving to the whole fruit a total anthocyanin content of 1.2 mg cyanidin 3-glucoside eq./g d.w. [15]. Black and Polignano carrots have already been characterized, showing a high total anthocyanin content (13.8 and 5.6 mg cyanidin 3-glucoside eq./g d.w., respectively), as well as a high level of phenolic acids, mostly chlorogenic acid (7.5 and 4.5 mg/g d.w, respectively) [15,16]. Polignano carrots are present as a local landrace, thus showing variable phenotypes. In this study, two different phenotypes (referred as phenotypes 1 and 2) were considered, with phenotype 2 being much richer than 1 in terms of anthocyanins (7.8 vs. 5 mg cyanidin 3-glucoside eq./g d.w.) and carotenoids (around tenfold more) [16].

Results of the AC assessment are summarized in Table 1. Under our experimental conditions, comparison of AC values of all tested extracts, as obtained by means of the LOX-FL and TEAC measurements, highlighted the superiority of the anthocyanin-rich extract from black carrot. AC values of the anthocyanin fraction of both types of Polignano carrot and ‘Sun Black’ tomato followed in the ranking. On the other hand, the ORAC method assessed the highest AC values in black carrot and ‘Sun Black’ tomato extracts, followed by Polignano carrots. All three AC assays agreed in detecting the lowest activities in lipophilic antioxidant-rich extracts from durum wheat whole flour.

When comparing different extracts (phenolic vs. lipophilic) from the same plant food, all three AC assays highlighted the expected higher antioxidant properties of durum wheat insoluble-bound phenolic fraction, known as the main antioxidant component in cereal grains [22], with respect to the lipophilic component; this is in accordance with our previous studies [18,21,23]. Concerning wildtype tomato, the LOX-FL assay showed much higher AC values in the lipophilic component than in the phenolic fraction. The opposite result was obtained by the ORAC measurement, while the TEAC assay did not highlight significant differences between the two extracts.

Interesting results were obtained through a comparison of AC values of the same extract from different plant foods. In this regard, when comparing lipophilic antioxidants from tomato and durum wheat whole meal, results are in agreement with those reported by Soccio et al. [18], showing much higher AC of tomato extracts than durum wheat, as evaluated by all three AC assays. This is in accordance with the high total carotenoid content (185 ± 0.4 μg/g d.w.) of tomato extracts measured in the present study, as well as with the high carotenoid and tocol contents already reported in previous studies [24,25]. Concerning the polyphenolic extract of tomato, the LOX-FL method agreed with the other two AC assays in pointing out the expected superiority of the ‘Sun Black’ genotype compared to the wildtype. The higher AC values for ‘Sun Black’ as compared to wildtype can be associated with a higher polyphenolic content, particularly anthocyanins [15]. As for carrot, all three AC assays agreed in measuring a higher AC value of the anthocyanin-rich extract from black carrot compared to the Polignano ones, likely attributable to the higher total anthocyanin and chlorogenic acid contents [16]. Interestingly, LOX-FL and TEAC, but not ORAC, showed the high antioxidant properties of Polignano carrot type 2 compared to the phenotype 1; this in accordance with the much higher content in anthocyanins of type 2.

### 2.2. Evaluation of Effects of Plant Food Extracts on h-GLYI and DWM-GLYI Activities

Exploration of biological activity of the different extracts under study was also carried out by evaluating their effect on GLYI enzymatic activity.

**Figure 1:**
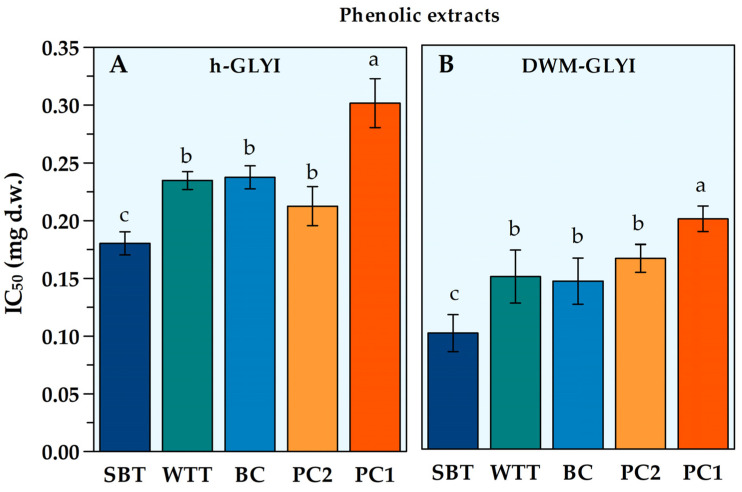
IC_50_ values for phenolic extracts from different plant foods, as obtained by both h-GLYI (**A**) and DWM-GLYI (**B**) assays. Data are reported as the mean value ± SD (*n* = 3). Different lowercase letters indicate significant differences at the *p* = 0.05 level, according to Duncan’s test. Abbreviations: SBT, ‘Sun Black’ tomato; WTT, wildtype tomato; BC, black carrot; PC1, Polignano carrot type 1; PC2, Polignano carrot type 2.

Firstly, the performance of the h-GLYI assay was tested. No modulating effect on h-GLYI assay was observed by the insoluble-bound phenolic extract from durum wheat in the tested range (0.05–1.0 mg d.w.). On the other hand, an inhibitory effect on h-GLYI reaction rate was observed in the presence of the polyphenolic extracts of both the wildtype and the purple-colored ‘Sun Black’ tomato fruits, as well as from the black carrot and from the two types of the purple-pigmented Polignano carrots. For quantitative measurement of the potency of extracts in inhibiting h-GLYI enzymatic activity, the amount of extract with half of maximal inhibitory effect (IC_50_) was calculated from the typical sigmoidal dose–response curve. IC_50_ values, expressed in terms of mg (d.w.) of food sample, are reported in Figure 1A.

As indicated by the lowest IC_50_ value, the anthocyanin fraction from ‘Sun Black’ tomato showed the highest efficacy in inhibiting h-GLYI activity, followed by wildtype tomato, black carrot, and Polignano carrot type 2, showing comparable inhibitory potency. Anthocyanins extracted from Polignano carrot type 1 appeared to be the least effective inhibitors of h-GLYI, showing a ~1.7-fold higher IC_50_ value compared to that of ‘Sun Black’ tomato.

The different anthocyanin-rich extracts were also studied with respect to their capability to modulate GLYI activity of highly purified mitochondrial fraction obtained from durum wheat (DWM-GLYI) [14]. As already reported for h-GLYI assay, an inhibitory effect was obtained in the presence of all anthocyanin-rich extracts under study. The corresponding IC_50_ values are reported in Figure 1B. Interestingly, a similar behavior to h-GLYI was obtained. Wildtype tomato, black carrot, and Polignano carrot type 2 extracts showed a similar inhibitory potency. The least effective inhibitors of DWM-GLYI activity were anthocyanins extracted from Polignano carrot type 1, showing a ~2-fold lower inhibitory efficiency than that of ‘Sun Black’ tomato, representing the extract with the highest capacity in inhibiting DWM-GLYI activity.

**Figure 2:**
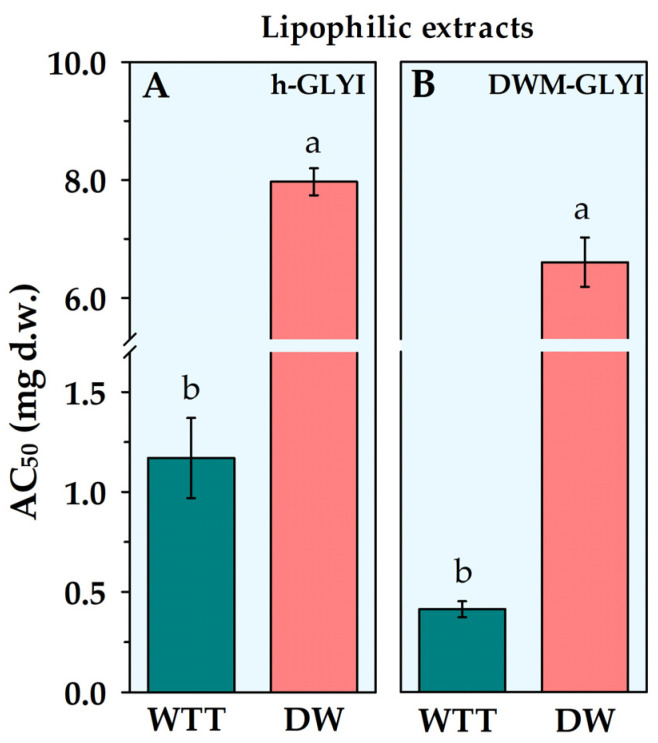
AC_50_ values for lipophilic extracts from wildtype tomato (WTT) and durum wheat (DW) whole grain, as obtained by both h-GLYI (**A**) and DWM-GLYI (**B**) assays. Data are reported as the mean value ± SD (*n* = 3). Different lowercase letters indicate significant differences at the *p* = 0.05 level, according to Duncan’s test.

Interestingly, extracts containing lipophilic antioxidant compounds from wildtype tomato, as well as from durum wheat grains, were found to induce an increase in h-GLYI reaction rate. In both cases, a linearly dependent relationship of activation with extract amount was obtained in the tested range. In order to allow a comparison among activating extracts, a new parameter was defined, representing the extract amount, expressed as mg (d.w.) of food sample, required for obtaining a 50% increase in GLYI activity (AC_50_). AC_50_ values relative to the h-GLYI assay are reported in Figure 2A, showing a ~7-fold higher activator potency of the lipophilic compounds from tomato fruits than from durum wheat whole flour.

Similarly, lipophilic compounds extracted from tomato and durum wheat grains were found to induce an increase in DWM-GLYI activity. AC_50_ values are reported in Figure 2B. The lipophilic extract of durum wheat whole flour showed a much lower activating efficacy than tomato, resulting about 17 times lower than that measured for tomato extracts.

### 2.3. Comparison of Performance of DWM-GLYI and h-GLYI Assays

Performance of DWM-GLYI system with respect to the evaluation of biological activity of plant food extracts was compared to that of h-GLYI. To this purpose, in Table 2, both IC_50_ and AC_50_ values obtained by DWM-GLYI assay for the different tested extracts are reported in comparison to those measured on h-GLYI.

It should be stressed that a lower extract amount able to induce a half modulation indicates a higher modulation efficiency and a more bioactive extract; this also implies that a lower IC_50_ or AC_50_ value indicates a higher responsivity to the extract of the biological system used to evaluate modulation. In light of these considerations, the comparison of IC_50_ and AC_50_ values between the two GLYI assays clearly shows the higher efficacy of both inhibiting and activating extracts in modulating the DWM-GLYI system than h-GLYI. To facilitate a comparison of the two systems with respect to the sensitivity to modulation by plant food extracts, the percentage decrease in IC_50_ or AC_50_ values relative to DWM-GLYI with respect to h-GLYI values was calculated for each extract under study. Interestingly, a general higher responsivity of the DWM-GLYI system was found with respect to the purified h-GLYI enzyme, up to about +65% in the case of extracts able to induce an increase of enzymatic activity, and up to about +45% for phenolic extracts exerting an inhibitory effect.

## 3. Discussion

The present study proposes a new in vitro approach to investigate the biological activity of antioxidant-rich plant extracts by evaluating their potential effect on both oxidative and dicarbonyl stresses. To this aim, the radical-scavenging properties of extracts were investigated by means of AC measurements, while the capability to affect RCS scavenging was evaluated by studying in vitro modulation of GLYI enzymatic activity, the major route for dicarbonyl detoxification.

To study different aspects of antioxidant action comprehensively, in vitro AC measurements of plant extracts were carried out using three assays (LOX-FL, ORAC, and TEAC) based on different chemical/biochemical principles. It should be outlined that, in this study, for the first time, the LOX-FL assay was applied to AC evaluation of anthocyanin-rich genotypes, such as ‘Sun Black’ tomato, as well as black and Polignano carrots. From a methodological point of view, AC results obtained in this study confirm the compelling properties of the LOX-1-based assays reviewed by Soccio et al. [18] as advisable tools for AC measurement of a large variety of plant foods, with high ability to discriminate among different samples and extracts and to provide a reliable AC information related to antioxidant content. Taken together, results of AC assessment performed in the present study are of some interest. They confirm the well-known antioxidant properties of the insoluble-bound phenolic component of durum wheat grain [18], as well as of the carotenoid-rich fraction of tomato [24,25]. In addition, these results highlight the excellent antioxidant potential of the unusual plant matrices being studied in this paper, such as ‘Sun Black’ tomato and anthocyanin-rich black and Polignano carrots, about which, so far, only few studies can be retrieved from literature [15,16,26,27].

Regarding GLYI assay, it was performed using the commercially available h-GLYI isoform, in comparison with the recently demonstrated and characterized DWM-GLYI activity. While AC measurements showed the ability of all investigated matrices to counteract oxidative stress, results of GLYI enzymatic assays highlighted different modes of modulation by plant extracts. No effect on both h-GLYI and DWM-GLYI assays was observed by the insoluble-bound phenolic extract from durum wheat in the investigated range. On the other hand, an inhibition on both GLYI systems was exerted by phenolic extracts of wildtype and ‘Sun Black’ tomatoes, as well as of black carrot and the two Polignano carrot types. The most and least effective inhibitors of GLYI activity resulted ‘Sun Black’ tomato and Polignano carrot type 1 extracts, respectively. On the contrary, lipophilic extracts from wildtype tomato and durum wheat grains induced an increase in GLYI activity, with a much higher efficacy of tomato than durum wheat.

The results of GLYI experiments allow some interesting observations. Firstly, both h-GLYI and DWM-GLYI assays appear to be suitable tools for studying the bioactive properties of antioxidant-rich food extracts, represented by their ability to exert either no effect or an activating or inhibiting action on the GLYI reaction. All tested extracts showed the same properties and ranking in modulation of h-GLYI and DWM-GLYI activities. Moreover, both GLYI activity systems showed the ability to discriminate among extracts of the same kind obtained from different plant sources, as well as among different types of extract obtained from the same plant food. Nevertheless, a higher sensitivity of the DWM-GLYI system to both inhibiting and activating extracts than the purified h-GLYI enzyme was observed. This could depend on the advantage of the “native” DWM-GLYI-based system compared to the purified recombinant enzyme to gain more biologically relevant insights on GLYI modulation by plant food extracts. In fact, in this case, evaluation was performed under experimental conditions resembling those observed in vivo, i.e., by studying interactions among GLYI, substrate, and plant food extract within the enzyme’s biological environment (subcellular organelles). These results are also in line with our previous studies, showing DWM as a good system for studying the physiological role and modulation of “native” plant sirtuins [28], as well as a very sensitive tool for evaluating the protecting/recovering action of phytochemicals against H_2_O_2_-induced damage to mitochondrial aconitase [29].

Interestingly, results obtained on both h-GLYI and DWM-GLYI systems strongly suggest that the different behavior of the tested plant food extracts can be strictly dependent on different chemical properties of the main different classes of phytochemicals contained in the extracts. In particular, our results indicate anthocyanins from the different tested plant sources as potent inhibitors of both h-GLYI and DWM-GLYI. As for DWM-GLYI activity, this result is in line with the inhibition already observed by curcumin (Ki = 20 μM) and quercetin (Ki = 55 μM) [14]. Similarly, our result relative to h-GLYI is in agreement with the potent inhibitory effect exerted by curcumin (Ki = 5.1 μM) and the flavonoids quercetin, myricetin, kaempferol, luteolin, and rutin (Ki values of 23, 13, 21, 35, and 140 μM, respectively) on the activity of GLYI purified from human erythrocytes, as demonstrated by Santel et al. [30]. The in vitro potent inhibitory effect of natural flavonoids possessing coplanar C-4 keto and C-5 hydroxyl groups (crucial for zinc chelation behavior), including myricetin, quercetin, luteolin, baicalein, and kaempferol (IC_50_ values of 0.56, 3.2, 7.7, 11.0 and 20.6 μM, respectively) was also demonstrated on purified recombinant h-GLYI enzyme by Takasawa et al. [31]. The same authors also showed the inhibitory abilities on h-GLYI activity of the flavonoid-similar structure compounds, anthocyanidins, such as delphinidin, cyanidin, and pelargonidin, with delphinidin being the most potent and selective GLYI inhibitor (IC_50_ = 1.9 μM) in this series, able to significantly suppress human leukemia HL-60 cell growth [32]. Trans-stilbene compounds were also identified as GLYI inhibitors, among which piceatannol is the most potent (IC_50_ =0.76 μM), also showing the ability to inhibit the proliferation of human lung cancer NCI-H522 cells [33].

Concerning the lack of effect of ferulic-rich extract from durum wheat grain observed in the present study, no information can be retrieved from the literature regarding the possible direct modulation of GLYI enzymatic activity by phenolic acids. To the best of our knowledge, the only available data regard the significantly increased levels of GLYI activity induced in rat insulin-secreting cell line (INS-1 832/13) by 48 h treatment with (50–100 μM) isoferulic acid [34]. The capability of this phenolic acid to protect the loss of GLYI activity in a short (0.5–2 h) period of MG exposure was also shown [34].

Compared to anthocyanins, in the present study, an opposite behavior was observed for lipophilic antioxidants compounds, such as tocols and carotenoids, extracted from tomato fruits and durum wheat grains, which were found to function as enzymatic activators of both h-GLYI and DWM-GLYI systems. To the best of our knowledge, very few literature studies concern the impact of lipophilic compounds on GLYI function. However, these studies provide controversial results, without assessment of direct activation of the GLYI enzyme. In particular, Suh et al. [35] showed that pretreatment of osteoblastic MC3T3-E1 cells with limonene (0.01–1 μM), a cyclic monoterpene present in the oil of citrus fruit peels, prior to 48 h MG exposure, induced a partial restoration of GLYI activity inhibited by MG, thus suggesting the limonene ability to scavenge MG protein adducts. On the other hand, vitamin D supplementation in type-2 diabetes participants was found to induce no significant increase in GLYI expression, whereas dietary vitamin E intake did not change mRNA GLYI levels in the brain of inbred strains of mice [10].

Actually, there are several studies on the hydrophilic/phenolic natural compounds acting as activators of GLYI function. Most of these data refer to the capability of this type of phytochemicals to increase GLYI function due to an enhanced gene expression via activation of the nuclear factor erythroid 2-related factor 2 (Nrf2)/antioxidant-response element (ARE) signaling pathway, as obtained by both in vitro (in several cellular types) and in vivo human/animal studies upon oral administration [10,12]. With respect to this point, in contrast to the ability of phenols to act as direct inhibitors of GLYI enzyme, there are many studies reporting their ability to activate GLYI gene expression. Resveratrol, fisetin, quercetin, genistein, and mangiferin are known to upregulate GLYI expression, and the binary combination of trans-resveratrol and hesperetin was also proven to synergistically increase GLYI expression and activity [10,12].

It should be outlined that, in addition to pure natural bioactive compounds, few studies have reported the evaluation of the effect on GLYI expression/activity of some plant extracts used in Asian traditional medicine. Monascin, a metabolite obtained from *Monascus*-fermented products, was found to enhance GLYI expression through Nrf2 activation and to protect from glycation stress rats orally administrated with MG [36]. Extracts from *Psoralea corylifolia* seeds increased the expression of hepatic GLYI in MG-treated mice [37]. Indole-4-carboxaldehyde isolated from the edible seaweed *Sargassum thunbergii* was also found to enhance expression of GLYI in human hepatocyte HepG2 cell line [38].

In light of the above discussion, the novelty of the present paper is its primary evaluation of the properties of a complex mixture of dietary antioxidant compounds to modulate GLYI enzymatic activity by directly interacting with the enzyme, performed in experimental conditions excluding any effect on gene expression. In particular, the ability of bioactive compounds to bind the GLYI enzyme and to enhance its activity is demonstrated for the first time, since to date only GLYI enzymatic inhibitors and no activators have been identified among pure natural compounds [10,12].

Our data provide other interesting elements for discussion. It should be underlined that the potential antiglycation function of natural bioactive products can involve many different mechanisms, mainly including inhibition of ROS damage (via reduced generation or increased ROS trapping), inhibition of MG formation (via MG trapping), activation of MG detoxification via activation of GLYI expression, and inhibition of harmful AGE formation. In this context, based on our results, lipophilic antioxidants are expected to exhibit antiglycation action, associated with their antioxidant properties and their capability to function as enzymatic activators of GLYI, the major MG-scavenging system. On the other hand, the behavior with respect to dicarbonyl stress is more complex in the case of phenolic/anthocyanin-rich products. Their excellent ability to scavenge ROS is accompanied by the property to strongly inhibit GLYI enzymatic activity, as pointed out in the present study; nevertheless, flavonoids are also known as GLYI expression inducers and inhibitors of AGE formation [39].

Discovery of extracts/phytochemicals with h-GLYI-activating or -inhibiting properties has very interesting physiological implications. Identification of natural bioactive compounds able to increase GLYI activity and/or expression (GLYI inducers), thus contributing to protection against MG accumulation and dicarbonyl stress, is receiving great interest for their very promising value as novel pharmaceuticals in prevention and treatment of type 2 diabetes, vascular complications of diabetes, and other AGE-promoted human disorders [12,40]. On the other hand, studies aimed at searching GLYI inhibitors may be equally worthwhile as a prospective treatment for cancer, since these inhibitors may induce severe dicarbonyl stress, allowing increased levels of AGEs to act as pro-apoptotic agents in cancer cells, particularly those showing high GLYI expressing-related multidrug-resistance [12,40].

In order to alleviate the detrimental effect of glycative stress with age and to counteract the progression of age- and dicarbonyl-related diseases, every strategy capable of prolonging the functionality of anti-AGE pathways with age should produce health beneficial effects. In the light of this, results of the present study indicate the GLYI assay as an advisable tool for searching plant foods as a safe and low-cost source of natural GLYI activity enhancers, whose consumption can be combined with pharmacological interventions aimed at extending the activity of the glyoxalase detoxifying route. On the other hand, the discovery of plant foods as a source of natural antioxidant compounds acting as GLYI enzymatic inhibitors is of equally remarkable significance for dietary management associated to the treatment of diseases related to inflammation and of multidrug-resistant tumors.

## 4. Materials and Methods

### 4.1. Chemicals and Plant Materials

All reagents at the highest commercially available purity were purchased from Sigma-Aldrich/Merck KGaA affiliates (Darmstadt, Germany). Certified seeds of durum wheat (*Triticum durum* Desf., cv Ofanto) were kindly supplied from the CREA-Cereal Research Center (Foggia, Italy). Tomato (*Solanum lycopersicum* L.) fruits (‘Sun Black’, with purple fruit, and the corresponding wildtype, with red fruit) were provided by Prof. A. Mazzucato (University of Tuscia, Viterbo, Italy). Black carrots (*Daucus carota* L. ssp. *sativus* var. *atrorubens* Alef.) were provided by ‘Aureli Mario’ farm (Ortucchio, L’Aquila, Italy). Purple Polignano carrots (a multi-colored landrace of *D. carota* L. ssp. *sativus*) were provided by small holder farmers of the area near Polignano a Mare (Bari, Italy).

### 4.2. Preparation of Plant Food Extracts

#### 4.2.1. Extraction of Lipophilic Compounds from Durum Wheat Whole Flour and Wildtype Tomato Fruits

Lipophilic compounds from durum wheat were extracted from daily milled whole flour according to the procedure described in Laus et al. [21] with minor modifications. Briefly, whole wheat flour (2 g) was saponified with 60% (*w*/*v*) KOH under nitrogen at 70 °C for 45 min, and then the suspension was extracted twice with 15 mL of *n*-hexane/ethyl acetate (9:1, *v*/*v*). The organic phases were collected, combined and evaporated to dryness under vacuum at 40 °C using Buchi R-215 Rotavapor System.

Lipophilic extracts from wildtype tomato fruits were prepared as reported in Pellegrini et al. [41] with some modifications. After washing and cutting, fruits were pooled, mixed, and homogenized under nitrogen in a high-speed blender. The homogenized sample (1 g) was suspended in 20 mL of acetone, stirred for 1 h in a melting ice bath, and then centrifuged at 30,000× *g* for 30 min at 4 °C. The supernatant was recovered and evaporated to dryness under vacuum at 40 °C.

Both wheat and tomato lipophilic extracts were reconstituted in ethanol and stored at −20 °C until analysis.

#### 4.2.2. Extraction of Phenolic Compounds from Durum Wheat Whole Flour, Tomato Fruits and Carrots

Insoluble-bound phenolic extracts from durum wheat were obtained as described in Laus et al. [21]. Briefly, the residual pellet from 80% (*v*/*v*) ethanol extraction of whole flour samples (1 g) was digested with 20 mL of 2 M NaOH at room temperature for 1 h. The resultant hydrolysate was acidified to pH 5–6 with acetic acid and centrifuged at 5000× *g* for 15 min at 20 °C; the supernatant was retained. The residue was washed twice with 10 mL of water and centrifuged at 5000× *g* for 10 min at 20 °C. The combined supernatants were concentrated under vacuum at 40 °C, adjusted to pH 2–3 with 6 M HCl, and extracted twice with *n*-hexane (at a *n*-hexane/water phase ratio equal to 1:1). The water phases were combined and extracted three times with ethyl acetate (at an ethyl acetate/water phase ratio equal to 1:1). The organic phases were pooled and evaporated to dryness under vacuum at 40 °C and the dry residue was reconstituted in 1 mL of water.

Polyphenolic-rich extracts from ‘Sun Black’ and wildtype tomatoes, as well as from black and purple Polignano carrots were obtained as reported in Blando et al. [15,16]. Briefly, 100 mg of freeze-dried material were macerated overnight at 4 °C in 10 mL of methanol/ethanol/water/formic acid (35:35:28:2 *v*/*v*/*v*/*v*) and centrifuged at 3500× *g* for 10 min. After centrifugation, the extraction was repeated on a rotary shaker at room temperature for 1 h. Supernatants were combined and organic solvent was evaporated in vacuum at 32 °C,, and the dry residue was reconstituted in acidified water (0.5% formic acid). All phenolic extracts were stored at −20 °C until analysis.

### 4.3. In Vitro Evaluation of Antioxidant Capacity of Plant Food Extracts Using TEAC, ORAC, and LOX-FL Methods

#### 4.3.1. TEAC Method 

The TEAC protocol, reported in Re et al. [20] and modified as in Laus et al. [21], was applied. The 2,2′-azinobis-(3-ethylbenzothiazoline-6-sulphonate) radical cation (ABTS^●+^) solution was produced by oxidation of ABTS aqueous solution with potassium persulfate. Before use, ABTS^●+^ was diluted in either 5 mM sodium phosphate buffer pH 7.4 or ethanol for AC determination of phenolic or lipophilic extracts, respectively, in order to obtain an absorbance at 734 nm (A_734_) of 0.70 ± 0.20. Then, 1.0 mL of diluted ABTS^●+^ solution was added with the extract and an appropriate volume of sodium phosphate buffer pH 7.40 (or ethanol) to obtain a final volume of assay mixture equal to 1.1 mL. Absorbance was measured exactly 5 min or 4 min (for phenolic or lipophilic extracts, respectively) after the extract addition. The percentage decrease in A_734_ of the samples with respect to A_734_ of the uninhibited radical cation solution was used to quantify AC.

#### 4.3.2. ORAC Method

ORAC protocol described in Ou et al. [19], using the 3′,6′-dihydroxyspiro[isobenzofuran-1[3H], 9′[9H]-xanthen]-3-one (fluorescein, FL) as a probe, and modified as reported in Laus et al. [21], was applied. The assay mixture (2 mL), preincubated at 37 °C for 15 min, contained 75 mM sodium phosphate buffer pH 7.40 and 6.3 nM FL; the reaction was started by adding 38 mM 2,2′-azobis(2-amidinopropane) (AAPH). FL fluorescence intensity decay was monitored at 37 °C by means of a Perkin Elmer LS-55 fluorescence spectrometer (λ_ex_ 485 nm; λ_em_ 515 nm). Measurements were carried out in the absence (blank) and presence of the tested extract. For AC determination of lipophilic extracts reconstituted in ethanol, a constant volume of ethanol was maintained in the assay mixture. To quantify AC, the area under the fluorescence decay kinetic curve was used, subtracted from that of the blank.

#### 4.3.3. LOX-FL Method

The LOX-FL reaction was performed as described in Soccio et al. [17], by continuously monitoring at 37 °C using a Perkin Elmer LS-55 fluorescence spectrometer the FL quenching (λ_ex_ 485 nm; λ_em_ 515 nm) coupled to linoleate hydroperoxidation by soybean LOX-1 isoform. The reaction mixture (2 mL) contained 100 mM sodium borate buffer pH 9.0, 400 μM sodium linoleate, 1 μL Tween 20/μmol linoleate, and 6.3 nM FL; the reaction was started by adding 0.5 EU of soybean LOX-1. LOX-FL reactions were carried out in the absence (control) or presence of the tested extract. Since the rate of the LOX-FL reaction is affected by ethanol, a constant volume of ethanol was maintained in the assay mixture for AC assessment of lipophilic extracts. The rate of the reaction was calculated as the highest slope to the experimental curve. The inhibition of the LOX-FL reaction was determined by calculating the percentage decrease in the rate of the FL quenching measured in the presence of the extract with respect to the control.

For all three AC methods, measurements were carried out in triplicate for at least three different amounts of extract, and AC was quantified by means of a dose–response curve obtained with (±)-6-hydroxy-2,5,7,8-tetramethylchromane-2-carboxylic acid (Trolox) as a standard antioxidant.

### 4.4. Isolation of Durum Wheat Mitochondria (DWM)

DWM were purified from a 48 h old etiolated seedling, as reported in Soccio et al. [42]. Briefly, durum wheat seeds (250–400 g), cv. Ofanto were sown on a distilled water-saturated polyurethane foam sheet covered with Whatman filter paper. They were dark grown at 25 °C and 80–85% relative humidity for 48 h in a Heraeus HPS 1500 incubator, and early seedlings (shoot length of about 0.3 cm) were used to obtain mitochondria.

The grinding and washing buffers were (i) 0.5 M sucrose, 4 mM cysteine, 1 mM EDTA, 30 mM Tris-HCl pH 7.50, 0.1% (*w*/*v*) defatted bovine serum albumin (BSA), and 0.6% (*w*/*v*) polyvinylpyrrolidone (PVP)-360, and (ii) 0.5 M sucrose, 1 mM EDTA, 10 mM Tris-HCl pH 7.40, and 0.1% (*w*/*v*) defatted BSA, respectively. Washed mitochondria were subjected to an isopycnic centrifugation in a self-generating density gradient containing 0.5 M sucrose, 10 mM Tris-HCl pH 7.20 and 28% (*v*/*v*) Percoll (colloidal PVP coated silica) in combination with a linear gradient of 0% (top) to 10% (bottom) PVP-40 to obtain the purified mitochondrial fraction.

Mitochondrial protein content was determined by the method of Lowry modified according to Harris [43] using BSA as a standard.

### 4.5. Glyoxalase I Activity Assay

GLYI activity was monitored as described by Schmitz et al. [8] and modified by Soccio et al. [14], by following at 240 nm and 25 °C the conversion of HA to S-LG, using a Perkin Elmer Lambda 45 UV/VIS spectrometer. HA was preliminarily generated by spontaneously reacting for 30 min 12 mM MG and 0.95 mM GSH in the assay mixture (final volume 0.4 mL) consisting of 100 mM sodium phosphate buffer (pH 7.20). In the adopted experimental conditions, using a dissociation constant _s_ equal to 3.0 mM, the actual [HA] resulted equal to 0.75 mM, while [MG_free_] and [GSH_free_] were 11.25 and 0.2 mM, respectively. The assay was performed both on the commercial recombinant GLYI isoform (SRP6125—Sigma Aldrich) (h-GLYI) and on the DWM proteins (DWM-GLYI activity). The reaction was started with the addition of 0.2 μg of h-GLYI. For DWM-GLYI activity assay, 20–30 μg of mitochondrial proteins were generally used, preliminarily lysed by incubation for 10 min with 0.1% (*v*/*v*) Triton X-100. GLYI reactions were carried out in both the absence (control) and the presence of the tested extract. The reaction rate was calculated as the highest slope of the experimental curve. The modulating effect of extracts was calculated as the percentage change in the reaction rate measured in the presence of the extract with respect to the control. All measurements were performed in at least three independent experiments with three replicates each time.

### 4.6. Spectrophotometric Determination of Total Carotenoid Content

Quantification of total carotenoids in lipophilic extracts from wildtype tomato was carried out according to the procedure described by Lichtenthaler [44]. Briefly, the absorbance spectra of appropriate dilutions in 80% (*v*/*v*) acetone of the lipophilic extract were recorded in the VIS region. Carotenoid concentration (mg/mL) was calculated by means of a proper equation using a specific absorption coefficient at 470 nm equal to 198 mL·mg^−1^·cm^−1^, as well as specific correction factors for chlorophyll a and b content based on absorbance measurements at 663 and 647 nm.

### 4.7. Statistical Analysis

Statistical analysis was performed using the Statistica 7.0 Data Analysis Software System (StatSoft Inc. 1984-2004). The normal distribution of data of Table 1 and Figure 1 and Figure 2 was verified using the Shapiro–Wilk test, and homogeneity of variances was verified using Bartlett’s test. Data of Table 1 and Figure 1 and Figure 2 were submitted to a “one-factor” analysis of variance (ANOVA) model. Following ANOVA, the multiple comparison post hoc Duncan’s test was applied to assess differences between multiple group means at the *p =* 0.05 level of significance. Data of Table 2 were submitted to Student’s *t*-test at the *p =* 0.05 and 0.01 levels of significance.

## Figures and Tables

**Table 1:** Antioxidant capacity (AC) of phenolic and lipophilic extracts from different plant sources, as evaluated by LOX-FL, ORAC and TEAC methods.

Plant Food	Extract	AC (μmol Trolox eq./g d.w.)		
		LOX-FL	ORAC	TEAC
DW	Phenolic	3.10 ± 0.05 ^g^	24.15 ± 1.50 ^e^	9.80 ± 0.80 ^d^
DW	Lipophilic	0.52 ± 0.06 ^h^	1.58 ± 0.17 ^f^	0.25 ± 0.01 ^f^
WTT	Lipophilic	14.90 ± 0.40 ^e^	31.10 ± 2.00 ^d^	3.70 ± 0.03 ^e^
WTT	Phenolic	8.78 ± 0.19 ^f^	89.62 ± 1.79 ^c^	3.80 ± 0.06 ^e^
SBT	Phenolic	27.14 ± 0.92 ^d^	199.9 ± 16.1 ^a^	10.10 ± 0.71 ^d^
BC	Phenolic	221.2 ± 24.02 ^a^	199.1 ± 14.4 ^a^	52.40 ± 0.25 ^a^
PC1	Phenolic	39.32 ± 0.09 ^c^	113.1 ± 6.3 ^b^	19.51 ± 1.20 ^c^
PC2	Phenolic	81.25 ± 2.39 ^b^	113.4 ± 4.5 ^b^	29.13 ± 0.03 ^b^

Measurements were performed as described in Section 4. Statistical analysis of ORAC and TEAC values was performed using natural logarithmic (ln)-transformed data; as for the LOX-FL measurements, square root (sr)-transformed data were used. All data are expressed as the mean value ± SD (*n* = 3 independent experiments). In each column, different lowercase letters indicate significant differences at the *p* = 0.05 level, according to the post hoc Duncan’s test, following ANOVA. Abbreviations: DW, durum wheat; SBT, ‘Sun Black’ tomato; WTT, wildtype tomato; BC, black carrot; PC1, Polignano carrot type 1; PC2, Polignano carrot type 2.

**Table 2:** IC_50_ and AC_50_ of plant food extracts, as obtained by h-GLYI and DWM-GLYI assays. For each type of tested extract, IC_50_ and AC_50_ obtained by both h-GLYI and DWM-GLYI assays are reported, as well as the comparison of sensitivity of the two GLYI assays, expressed as the variation (%) of DWM-GLYI IC_50_ (or AC_50_) value with respect to the corresponding value obtained by h-GLYI assay.

Plant Food	Extract	IC_50_ or AC_50_ (mg d.w.)		
		h-GLYI	DWM-GLYI	DWM-GLYI/h-GLYI (% Variation)
DW	Lipophilic	7.97 ± 0.23 ^a^	6.60 ± 0.42 ^a^	+17% **
WTT	Lipophilic	1.17 ± 0.20 ^a^	0.412 ± 0.040 ^a^	+65% **
WTT	Phenolic	0.235 ± 0.008 ^b^	0.150 ± 0.023 ^b^	+36 **
SBT	Phenolic	0.180 ± 0.010 ^b^	0.101 ± 0.016 ^b^	+44% **
BC	Phenolic	0.237 ± 0.010 ^b^	0.146 ± 0.020 ^b^	+37% **
PC1	Phenolic	0.302 ± 0.021 ^b^	0.200 ± 0.011 ^b^	+34% **
PC2	Phenolic	0.213 ± 0.017 ^b^	0.165 ± 0.012 ^b^	+22% *

^a^ AC_50_; ^b^ IC_50_. Data are reported as the mean value ± SD (*n* = 3). * *p ≤* 0.05, ** *p ≤* 0.01, where *p* represents the probability level according to the Student’s *t*-test relative to the comparison between each DWM-GLYI IC_50_ (or AC_50_) with the corresponding h-GLYI value. Abbreviations: SBT, ‘Sun Black’ tomato; WTT, wildtype tomato; BC, black carrot; PC1, Polignano carrot type 1; PC2, Polignano carrot type 2.

## Data Availability

Not applicable.

## Abbreviations

AAPH, 2,2′-azobis(2-amidinopropane); ABTS, 2,2′-azinobis-(3-ethylbenzothiazoline-6-sulphonate); AC, antioxidant capacity; AC_50_, extract amount required for obtaining a 50% increase in enzyme activity; AGE, advanced glycation end-product; ARE, antioxidant-response element; BC, black carrot; BSA, bovine serum albumin; DW, durum wheat; DWM, durum wheat mitochondria; DWM-GLYI, glyoxalase I from DWM; FL, fluorescein; GLYI, glyoxalase I; GLYII, glyoxalase II; GSH, reduced glutathione; HA, hemithioacetal; HAT, hydrogen atom transfer; h-GLYI, human recombinant GLYI isoform; IC_50_, extract amount required to halve the enzyme activity; Ki, inhibition constant; LOX, lipoxygenase; MG, methylglyoxal; Nrf2, nuclear factor erythroid 2-related factor 2; ORAC, oxygen radical absorbance capacity; PC1, Polignano carrot type 1; PC2, Polignano carrot type 2; PVP, polyvinylpyrrolidone; RCS, reactive dicarbonyl species; ROS, reactive oxygen species; SBT, ‘Sun Black’ tomato; SET, single electron transfer; S-LG, S-D-lactoylglutathione; TEAC, Trolox equivalent antioxidant capacity; Trolox, (±)-6-hydroxy-2,5,7,8-tetramethylchromane-2-carboxylic acid; WTT, wildtype tomato.
